# Incidence and Predictors of Perioperative Cardiac Events in Emergency Abdominal Surgeries: A Retrospective Study

**DOI:** 10.7759/cureus.106613

**Published:** 2026-04-07

**Authors:** Swetha Srinivasan, Sai Madhavan Chandrasekar, Kannan Lakshminarayanan

**Affiliations:** 1 Medicine, Sri Ramachandra Institute of Higher Education and Research, Chennai, IND; 2 Community Medicine, Sri Ramachandra Institute of Higher Education and Research, Chennai, IND

**Keywords:** appendicectomy, cardiovascular risk, cholecystectomy, emergency surgeries, perioperative cardiac events, retrospective study

## Abstract

Background

Perioperative cardiac events are clinically significant complications in non-cardiac surgery and contribute to increased morbidity and prolonged hospitalization. Emergency abdominal surgeries pose additional challenges due to acute presentation, limited preoperative optimization, hemodynamic instability, systemic inflammation, and heightened sympathetic response. Region-specific data from Indian tertiary care centers are limited.

Objectives

This study aimed to determine the incidence of perioperative cardiac events in patients undergoing emergency appendicectomy or cholecystectomy and to identify patient-related predictors associated with the occurrence of these perioperative cardiac events.

Methods

A retrospective study was conducted among adult patients undergoing emergency appendicectomy or cholecystectomy at Sri Ramachandra Hospital between January 2015 and December 2025. Demographic details, cardiovascular comorbidities, type of surgery, perioperative cardiac events, and length of hospital stay were extracted from medical records. Cardiac events were classified as major or minor. Categorical variables were compared using Fisher’s exact test and continuous variables using the Mann-Whitney U test. A p-value <0.05 was considered statistically significant.

Results

A total of 272 adult patients were included in the study. Perioperative cardiac events occurred in eight patients (2.9%), comprising four major (1.45%) and four minor (1.45%) events. Major cardiac events included hypotension requiring inotropic support and one episode of acute myocardial infarction, while minor events consisted of transient arrhythmias and self-limiting hemodynamic disturbances. No perioperative mortality was recorded. Cardiac events were more frequent in patients aged ≥60 years and in those with pre-existing cardiovascular comorbidities. Patients who developed cardiac events had a longer median hospital stay compared to those without events (6.5 vs 4 days).

Conclusion

Perioperative cardiac events in emergency appendicectomy and cholecystectomy are uncommon but clinically meaningful. Advanced age and pre-existing cardiovascular comorbidities are key predictors of risk. Focused perioperative assessment and vigilant monitoring are essential to reduce morbidity in emergency abdominal surgery.

## Introduction

Perioperative cardiac complications remain a major contributor to morbidity and mortality following noncardiac surgery [1,2]. These complications arise from an imbalance between myocardial oxygen supply and demand due to surgical stress, sympathetic activation, inflammation, and hemodynamic fluctuations, which may lead to ischemia, arrhythmias, or heart failure [1,2]. Patients with pre-existing cardiovascular disease are particularly vulnerable, and perioperative hemodynamic instability and intraoperative management significantly influence cardiac outcomes [1,2]. To predict and reduce these risks, perioperative cardiovascular risk stratification tools such as the Revised Cardiac Risk Index (RCRI) have been developed and validated [3,4]. In addition, biomarkers, especially troponin, have improved detection of myocardial injury after noncardiac surgery (MINS), identifying clinically silent ischemic events associated with adverse long-term outcomes [5,6]. These findings have shaped international guidelines recommending preoperative cardiovascular evaluation and perioperative optimization in at-risk patients [7].

Although cardiac complications have been extensively studied in major abdominal and high-risk noncardiac surgery, evidence focusing specifically on emergency abdominal procedures such as appendicectomy and cholecystectomy is limited. Major abdominal surgery has been associated with significant cardiac morbidity, with earlier studies reporting cardiac complication rates of approximately 5.7% in elective abdominal surgery and even higher rates in urgent and high-risk cohorts [8,9]. Large multicenter prospective audits have demonstrated that postoperative cardiovascular complications significantly increase 30-day mortality after major abdominal surgery [10,11]. Urgent procedures carry a higher cardiac risk than elective surgeries due to limited time for preoperative optimization and increased physiological stress [12].

In abdominal surgery, myocardial injury and cardiac complications have been linked to intraoperative hypotension, vasopressor use, hypothermia, transfusion, and pre-existing arrhythmias such as atrial fibrillation [13,14]. Laparoscopic cholecystectomy induces hemodynamic changes due to pneumoperitoneum and CO₂ insufflation, which can precipitate arrhythmias and instability in susceptible patients [15]. Despite the high frequency of emergency appendicectomy and cholecystectomy, these procedures are often perceived as low-risk, resulting in limited cardiac monitoring and underrepresentation in perioperative cardiac research. However, systematic reviews of acute care surgery complications indicate that cardiac events, although rare, do occur after appendicectomy and cholecystectomy, particularly in patients with underlying risk factors [16,17].

Recent evidence suggests that cholecystectomy may influence long-term cardiovascular and metabolic outcomes, especially in patients with dyslipidemia, which may further impact perioperative cardiac risk [16,17]. However, there remains a lack of data on the incidence and predictors of perioperative cardiac events, specifically in emergency appendicectomy and cholecystectomy. Therefore, this study aims to evaluate the incidence and predictors of perioperative cardiac events in emergency abdominal surgeries at a tertiary care hospital, with the goal of improving perioperative risk stratification and monitoring strategies in this commonly performed but understudied surgical population [18-21].

The objectives were to determine the incidence of perioperative cardiac events in emergency appendicectomy and cholecystectomy and to identify patient-related predictors associated with the occurrence of these events.

We hypothesize that perioperative cardiac events, although infrequent, are significantly associated with advanced age and pre-existing cardiovascular comorbidities.

## Materials and methods

Study design and setting

This was a retrospective study conducted at Sri Ramachandra Hospital, Porur, Chennai, a tertiary care teaching hospital in South India.

Study period

The study included patients who underwent surgery between January 2015 and December 2025.

Inclusion criteria

Patients aged more than 18 years who underwent emergency appendicectomy or emergency cholecystectomy were included in the study.

Exclusion criteria

Patients undergoing elective surgical procedures and those under 18 years of age were excluded from the study.

Consecutive eligible patients meeting the inclusion criteria during the study period were included to minimize selection bias.

Ethical approval

The study was approved by the Institutional Ethics Committee of Sri Ramachandra Institute of Higher Education and Research (IEC number: CSP-III/25/OCT27/406). A waiver of informed consent was granted due to the retrospective nature of the study and the absence of direct patient contact.

Data collection

Patient data were retrieved from hospital medical records using unique case identifiers. The following variables were extracted:

Age and sex;

cardiovascular comorbidities, including hypertension, coronary artery disease, and dyslipidemia;

type of surgery (appendicectomy or cholecystectomy);

surgical approach (laparoscopic or open);

American Society of Anesthesiologists (ASA) physical status;

duration of surgery;

occurrence and type of perioperative cardiac events;

length of hospital stay.

Data collection was performed using a standardized data collection form by reviewing electronic medical records to ensure consistency.

Definitions

Perioperative cardiac events were defined as cardiovascular complications occurring during the intraoperative period or postoperatively until hospital discharge. Pre-existing cardiac abnormalities documented prior to surgery were considered risk factors and were not classified as perioperative cardiac events.

Perioperative cardiac events were further categorized as major or minor.

Major cardiac events included the following:

Acute myocardial infarction or acute coronary syndrome, defined as clinical symptoms with electrocardiographic changes and/or elevated cardiac biomarkers.

Hypotension requiring inotropic support, defined as persistent hypotension necessitating pharmacological vasopressor or inotropic therapy.

Significant arrhythmias requiring intervention, including tachyarrhythmias or bradyarrhythmias requiring medical or electrical treatment.

Minor cardiac events included the following:

Transient arrhythmias, such as self-limiting supraventricular tachycardia or sinus bradycardia, not requiring treatment.

Self-limiting electrocardiographic changes, including transient ST-T wave changes without clinical or biochemical evidence of ischemia.

Stable bradycardia or tachycardia, not requiring any therapeutic intervention.

Statistical analysis

Categorical variables were analyzed using Fisher’s exact test. Continuous variables were expressed as median values and compared using the Mann-Whitney U test. A p-value of less than 0.05 was considered statistically significant. Multivariate analysis was not performed due to the small number of cardiac events observed. Odds ratios were calculated to estimate the strength of association between selected predictors and perioperative cardiac events.

There were no missing data for the variables included in the analysis.

## Results

Patient characteristics

The study included 272 adult patients who underwent emergency abdominal surgery. The mean age of the study population was 35.9 ± 15.8 years (range: 18-86 years), with 27 patients (9.9%) aged ≥60 years. There were 171 male patients (62.9%) and 101 female patients (37.1%). Appendicectomy alone was performed in 233 patients (85.7%), cholecystectomy alone in 37 patients (13.6%), and both procedures during the same admission in two patients (0.7%). Patient’s baseline characteristics of the study population are summarized in Table 1.

**Table 1:** Patient characteristics of the study population (n = 272) SD, standard deviation; ASA, American Society of Anesthesiologists; IQR, interquartile range.

Variable	Value
Age, mean ± SD, years	35.9 ± 15.8
Age ≥60 years, n (%)	27 (9.9)
Sex, n (%)	
Male	171 (62.9)
Female	101 (37.1)
ASA physical status, n (%)	
ASA I-II	239 (87.9)
ASA III-IV	33 (12.1)
Type of surgery, n (%)	
Appendicectomy alone	233 (85.7)
Cholecystectomy alone	37 (13.6)
Both procedures	2 (0.7)
Duration of surgery, median (IQR), minutes	120 (90-150)

Surgical procedures

The distribution of surgical procedures and approaches is summarized in Table 2.

**Table 2:** Distribution of surgical procedures and approaches in the study population

Procedure	Laparoscopic	Open	Total
Appendicectomy	216	21	237
Cholecystectomy	34	8	42
Combined	2	0	2

Incidence of perioperative cardiac events

A total of eight patients (2.9%) experienced perioperative cardiac events. Among these are the following:

Major events: Four patients (1.45%) -- three hypotension requiring inotropes, one acute myocardial infarction/acute coronary syndrome.

Minor events: Four patients (1.45%) -- transient supraventricular arrhythmias, sinus bradycardia, self-limiting ECG changes.

All cardiac events occurred predominantly in the postoperative period, while preoperative abnormalities were considered risk factors. No perioperative mortality was recorded.

The median duration of surgery was longer in patients who developed perioperative cardiac events compared to those without events (148 minutes vs 115 minutes). However, causality cannot be established due to the retrospective nature of the study.

**Figure 1:**
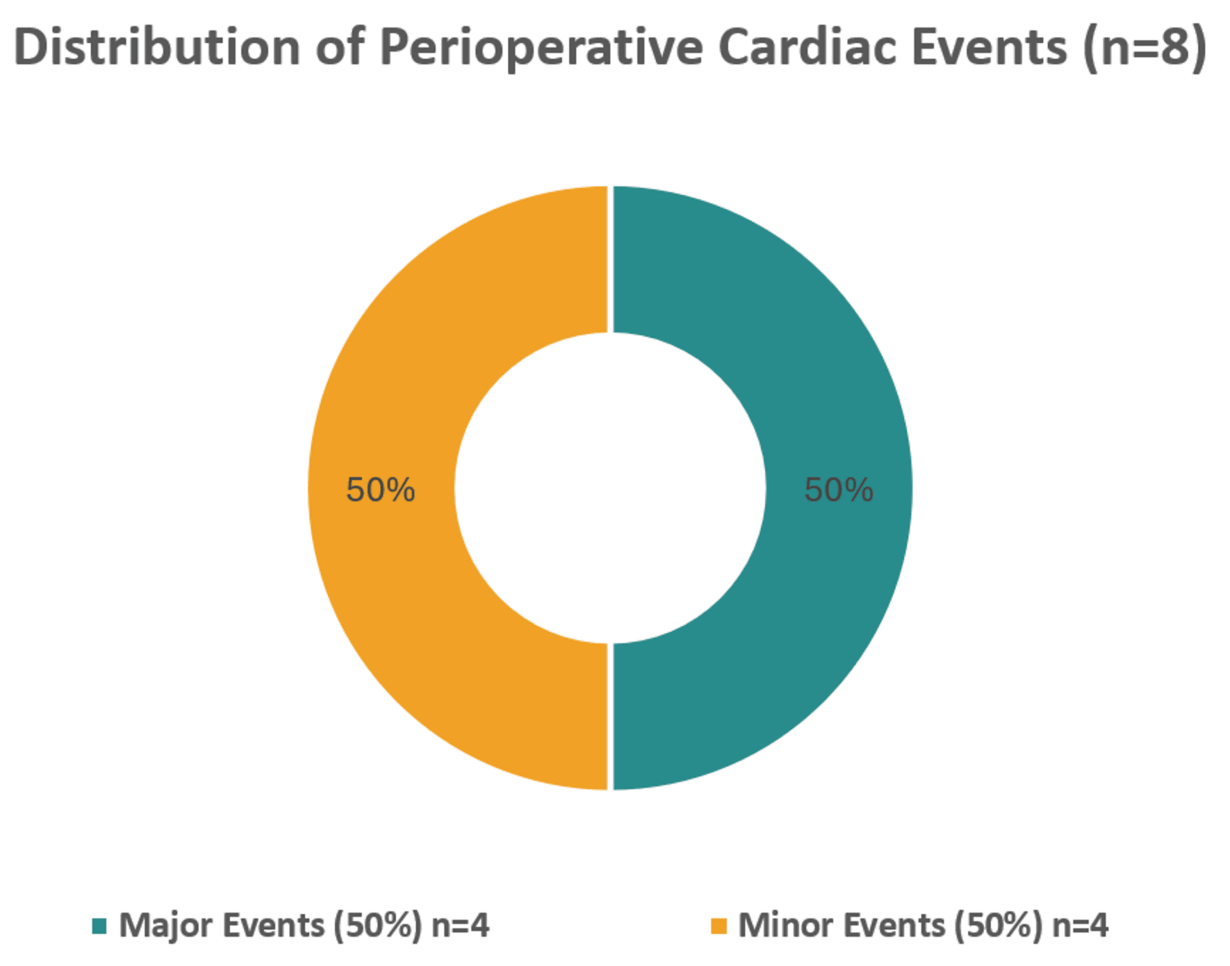
Distribution of perioperative cardiac events (n = 8)

The distribution of perioperative cardiac events is shown in Figure 1.

Characteristics of patients with perioperative cardiac events

Details of the event group, including preoperative, intraoperative, and postoperative findings, are shown in Table 3.

**Table 3:** Characteristics of patients with perioperative cardiac events (Event group)

Age	Sex	Surgery	Comorbidities	Pre-op Findings	Intra-op Events	Post-op Events	Event Severity
60	Male	Laparoscopic cholecystectomy	None	None	None	Recurrent supraventricular tachycardia	Minor
68	Male	Laparoscopic cholecystectomy	Coronary artery disease, left ventricular dysfunction	None	Hypertension	Postoperative hypertension	Minor
44	Male	Laparoscopic cholecystectomy	Hypertension, coronary artery disease, dyslipidemia	T-wave depression	None	Hypotension (inotropes)	Major
56	Female	Laparoscopic cholecystectomy	Hypertension, coronary artery disease	None	None	Acute coronary syndrome	Major
67	Female	Laparoscopic cholecystectomy	Hypertension, unstable angina	None	Hypotension	Continued inotropes	Major
48	Female	Laparoscopic appendicectomy	None	Diabetic ketoacidosis	None	Sinus bradycardia	Minor
53	Female	Laparoscopic appendicectomy	None	None	None	Mild coronary artery disease changes	Minor
67	Male	Open appendicectomy	Hypertension	None	Hypotension	Inotropes	Major

Perioperative cardiac events by type of surgery

The distribution of cardiac events by type of surgery is shown in Table 4.

**Table 4:** Perioperative cardiac events according to type of surgery

Type of Surgery	Patients With Cardiac Events	Total Patients	Event Rate (%)
Appendicectomy	3	237	1.3
Cholecystectomy	5	37	13.5

This demonstrates that although fewer patients underwent cholecystectomy, the proportion of cardiac events was higher in this group.

Age distribution and comorbidity profile

Patients aged ≥60 years accounted for four of eight cardiac events (50%), while only 4.4% of patients without events were ≥60 years.

**Figure 2:**
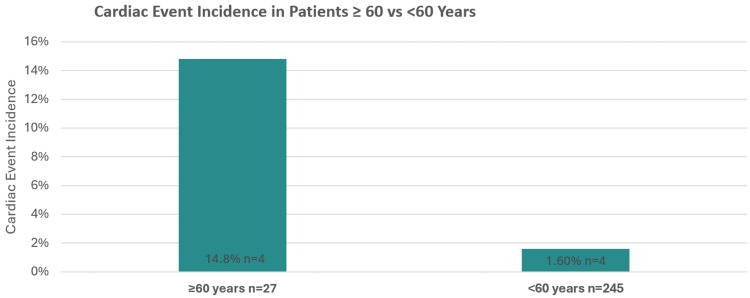
Cardiac event incidence in patients ≥60 vs <60 years

Comparison of cardiac event incidence in patients ≥60 vs <60 years is shown in Figure 2.

The comparison of age and cardiovascular comorbidities in event vs non-event groups is shown in Table 5.

**Table 5:** Comparison of age, cardiovascular comorbidities, and ASA in event vs non-event groups Statistical analysis: Fisher's exact test (two-tailed). Fisher's exact test does not produce a test statistical value; only the p-value is shown. ASA, American Society of Anesthesiologists.

Variable	Event Group n (%)	Non-event n (%)	p-Value	Odds Ratio
Age ≥60 years	4 (50%)	23 (8.7%)	0.004	10.5
Hypertension	3 (37.5%)	24 (9.1%)	0.035	6.0
Coronary artery disease	2 (25%)	3 (1.2%)	0.007	28.9
Dyslipidemia	1 (12.5%)	2 (0.7%)	0.048	18.7
ASA III-IV	3 (37.5%)	30 (11.4%)	-	4.7

Higher ASA grade (III-IV) was more frequently observed in patients with perioperative cardiac events. ASA III-IV was associated with increased odds of perioperative cardiac events (OR: 4.7). However, this association should be interpreted with caution due to the small number of events.

**Figure 3:**
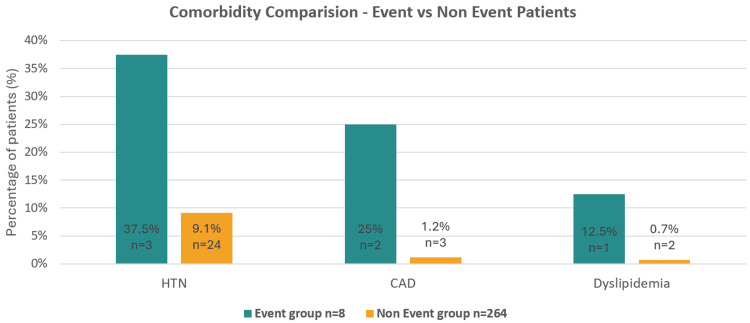
Cardiac comorbidity comparison -- Event vs non-event patients HTN, hypertension; CAD, coronary artery disease.

The comparison of cardiac comorbidities in event vs non-event groups is shown in Figure 3.

Length of hospital stay

Patients who experienced perioperative cardiac events had a significantly longer median hospital stay compared to those without events, reflecting increased perioperative morbidity and resource utilization (6.5 days vs 4 days; p < 0.001).

## Discussion

The incidence of perioperative cardiac events in our study was 2.9% among patients undergoing emergency appendicectomy and cholecystectomy, a figure that is consistent with the lower end of the incidence range reported in noncardiac surgery populations, which generally falls between 1% and 5% [1,2]. The observed incidence is particularly notable because it reflects an emergency surgical cohort, where physiological stress, inflammation, and hemodynamic instability are expected to be higher than in elective surgery [1,2]. Perioperative cardiac events are driven by sympathetic activation, increased myocardial oxygen demand, and inflammatory responses that can precipitate ischemia, arrhythmias, or hemodynamic collapse, especially in vulnerable patients [1,2]. While risk models such as the RCRI and other clinical risk indices are widely used for predicting cardiac risk in noncardiac surgery, their applicability to emergency abdominal surgeries remains less studied, and this study adds valuable data by directly evaluating perioperative cardiac outcomes in this specific emergency setting [3,4].

Biomarkers such as troponin have transformed the detection of MINS, uncovering clinically silent ischemic events that would otherwise remain undetected [5]. The BASEL-PMI study further emphasized that perioperative myocardial injury is associated with poor long-term outcomes, highlighting that even subclinical events are clinically significant [6]. These findings support the notion that cardiac events may be underreported in settings where routine biomarker screening is not performed. Nonetheless, the low incidence in our study suggests that emergency appendicectomy and cholecystectomy, despite being urgent procedures, may inherently carry lower cardiac risk than major abdominal surgery due to shorter operative duration, lower blood loss, and generally younger patient demographics.

Large cohort studies of major abdominal surgery have demonstrated higher rates of cardiac complications, particularly in urgent and high-risk cohorts [9]. Cardiac complication rates after elective abdominal surgery have been reported around 5.7%, with associated increases in length of stay and mortality [8]. Similarly, large international prospective studies have shown that postoperative cardiovascular complications significantly increase 30-day mortality after major abdominal surgery, emphasizing the clinical importance of recognizing and preventing these events [10,11]. Our study’s incidence is lower, which may be attributable to the emergency surgeries being relatively shorter and involving less physiologic insult compared with major abdominal resections. Yet, the presence of cardiac events in 2.9% of cases highlights that even routine emergency abdominal procedures are not free from cardiac risk, especially when patient comorbidities are present.

The association of age ≥60 years and cardiovascular comorbidities such as hypertension, CAD, and dyslipidemia with perioperative cardiac events in this study aligns with established perioperative cardiac risk predictors [3,4]. These comorbidities reflect the underlying burden of atherosclerotic disease and impaired cardiac reserve, which predispose patients to ischemia and arrhythmias during the stress of surgery. The ANESCARDIOCAT study also demonstrated that major adverse cardiac and cerebrovascular events in noncardiac surgery are more likely in patients with pre-existing cardiovascular disease, further supporting our findings [9]. Additionally, the higher risk associated with urgent surgery compared to elective procedures has been demonstrated in recent studies, showing increased cardiac event rates in urgent cohorts [12]. This supports the idea that emergency abdominal surgery, despite being generally less complex than major abdominal operations, still presents significant cardiac stress, especially in older or comorbid patients.

In our cohort, major cardiac events were predominantly hypotension requiring inotropic support and one case of acute myocardial infarction. This is consistent with evidence that intraoperative hypotension and hemodynamic instability are strong predictors of myocardial injury [13]. A South Indian study on high-risk abdominal surgery identified vasopressor use, hypothermia, and transfusion as predictors of MINS, reinforcing the importance of intraoperative hemodynamic stability [13]. The role of atrial fibrillation and arrhythmia burden in postoperative cardiovascular outcomes has also been documented, with pre-existing atrial fibrillation shown to increase postoperative mortality and cardiovascular complications after noncardiac surgery [14]. This supports the clinical relevance of even minor arrhythmias observed in our cohort, as arrhythmia burden may reflect underlying cardiac vulnerability.

Laparoscopic cholecystectomy can cause significant cardiovascular changes due to pneumoperitoneum, CO₂ insufflation, and sympathetic stimulation, which may predispose patients to arrhythmias and hemodynamic shifts [15]. These physiologic changes could explain the arrhythmias and hemodynamic instability seen in several cases, even in patients without major comorbidities. Moreover, systematic reviews of complications after appendicectomy and cholecystectomy show that cardiac complications, although rare, are recognized adverse events in acute care surgery [16,17]. This reinforces that cardiac monitoring and risk stratification are important even in commonly performed emergency procedures.

The length of hospital stay was significantly longer in patients with cardiac events (6.5 vs 4 days), which aligns with earlier evidence that perioperative cardiac complications prolong hospitalization and increase resource utilization [8]. This underscores the economic and clinical impact of cardiac events, even when mortality is low. Although no perioperative mortality occurred in our cohort, multicenter studies have demonstrated that postoperative cardiovascular complications significantly increase mortality after major abdominal surgery, indicating that cardiac events should not be underestimated [10,11].

Recent evidence suggests that cholecystectomy may influence long-term metabolic and cardiovascular risk, particularly in patients with metabolic comorbidities [16,17]. While these studies focus on long-term outcomes rather than immediate perioperative events, they underscore that patients undergoing emergency cholecystectomy may have underlying metabolic vulnerability that contributes to cardiovascular risk. In addition, reviews of perioperative cardiac complications and evidence-based strategies emphasize the importance of preoperative risk assessment, intraoperative monitoring, and postoperative surveillance, particularly in high-risk patients [18]. Large-scale analyses of cardiac complications in major abdominal surgery have shown increasing trends, emphasizing that cardiac risk remains an important consideration in abdominal surgery [19]. Enhanced recovery pathways and perioperative care guidelines also emphasize structured perioperative monitoring and early detection of complications, which may reduce cardiac morbidity in surgical patients [20]. Finally, evidence from tertiary care settings suggests that cardiac monitoring in older patients undergoing abdominal surgery can help identify perioperative cardiovascular events, reinforcing the need for targeted monitoring in high-risk groups [21].

This study has certain limitations. First, its retrospective design may introduce inherent biases related to data collection and documentation. Second, the relatively small number of perioperative cardiac events limits the ability to perform multivariate analysis and may affect the strength of statistical associations. Third, the study was conducted at a single tertiary care center, which may limit the generalizability of the findings to other settings. Despite these limitations, the study provides clinically relevant insights into perioperative cardiac risk in commonly performed emergency abdominal surgeries.

## Conclusions

Perioperative cardiac events following emergency appendicectomy and cholecystectomy are uncommon but clinically significant, particularly in elderly patients and those with pre-existing cardiovascular comorbidities. Although emergency settings limit opportunities for extensive preoperative optimization, early identification of high-risk patients, vigilant perioperative monitoring, and prompt management of hemodynamic disturbances can help reduce postoperative morbidity and hospital stay. Awareness of these risk factors is essential for surgeons and anesthesiologists managing emergency abdominal surgeries, especially in resource-limited settings.
